# Evaluating Dose- and Time-Dependent Effects of Vitamin C Treatment on a Parkinson's Disease Fly Model

**DOI:** 10.1155/2019/9720546

**Published:** 2019-01-02

**Authors:** Huynh Man Anh, Dao My Linh, Vuu My Dung, Dang Thi Phuong Thao

**Affiliations:** ^1^Department of Molecular and Environmental Biotechnology, Faculty of Biology and Biotechnology, University of Science, Vietnam National University Ho Chi Minh City, Ho Chi Minh City 700000, Vietnam; ^2^Laboratory of Molecular Biotechnology, University of Science, Vietnam National University Ho Chi Minh City, Ho Chi Minh City 700000, Vietnam

## Abstract

Parkinson's disease (PD) is a common neurodegenerative disorder and characterized by progressive locomotive defects and loss of dopaminergic neurons (DA neuron). Currently, there is no potent therapy to cure PD, and the medications merely support to control the symptoms. It is difficult to develop an effective treatment, since the PD onset mechanism of PD is still unclear. Oxidative stress is considered as a major cause of neurodegenerative diseases, and there is increasing evidence for the association between PD and oxidative stress. Therefore, antioxidant treatment may be a promising therapy for PD. *Drosophila* with knockdown of *dUCH*, a homolog of *UCH-L1* which is a PD-related gene, exhibited PD-like phenotypes including progressive locomotive impairments and DA neuron degeneration. Moreover, knockdown of *dUCH* led to elevated level of ROS. Thus, *dUCH* knockdown flies can be used as a model for screening of potential antioxidants for treating PD. Previous studies demonstrated that curcumin at 1 mM and vitamin C at 0.5 mM could improve PD-like phenotypes induced by this knockdown. With the purpose of further investigating the efficiency of vitamin C in PD treatment, we used *dUCH* knockdown *Drosophila* model to examine the dose- and time-dependent effects of vitamin C on PD-like phenotypes. The results showed that although vitamin C exerted neuroprotective effects, high doses of vitamin C and long-term treatment with this antioxidant also resulted in side effects on physiology. It is suggested that dose-dependent effects of vitamin C should be considered when used for treating PD.

## 1. Introduction

Parkinson's disease (PD) is one of the most common neurodegenerative disorders and impacts 1% of the population aged over 60 years [1]. PD is a progressive neurological disorder characterized by locomotive defects such as tremor, rigidity, and bradykinesia. In addition, this disease is also associated with nonmotor symptoms including hyposmia, sleep disorders, and depression [2, 3]. The neuropathological hallmarks of PD are the loss of dopaminergic neurons (DA neurons) in substantia nigra and the formation of Lewy bodies in the brain [4, 5]. The complex interactions between genetic and environmental factors are involved in the pathogenesis of PD. It has been reported that many genes and their variants such as *α-synuclein*, *PINK-1*, *DJ-1*, *UCH-L1*, and *LRRK2* are related to PD. However, onset mechanisms of PD have not yet been fully elucidated [6–8].

Oxidative stress is regarded as a major cause of neurodegenerative diseases including PD [9]. Many findings have provided the evidence of the link between oxidative stress and PD. The decreased level of reduced glutathione (GSH) and increased oxidative damage of lipid, protein, and DNA were found in the brains of PD patients [10–16]. Specially, there are many sources of free radicals in brain such as the high consumption of oxygen and glucose and neurotransmitter metabolism, leading to the susceptibility of brain to oxidative stress [17]. Furthermore, DA neurons themselves can generate oxidative stress through dopamine metabolism [18, 19]. Therefore, antioxidant treatment is considered as a promising therapy for PD [20]. Antioxidants such as curcumin, resveratrol, and vitamin E have been examined, and they exerted positive neuroprotective effects in models of PD [21–23].

Vitamin C is a well-known and essential antioxidant; however, humans are not able to synthesize vitamin C due to lack of enzyme L-gulonolactone oxidase. Therefore, this vitamin has to be obtained through diet and supplements [24]. Vitamin C performs many important biological functions. For example, it can act as an antioxidant scavenging free radical and reactive oxygen species and cofactor of enzyme [25]. It is of note that vitamin C is concentrated highly in brain. In the nervous system, besides playing an antioxidant role, vitamin C is also involved in different processes including neuronal differentiation, maturation and survival, catecholamine biosynthesis (such as dopamine and norepinephrine), and modulation of neurotransmission [26]. Studies on PD patients reported that vitamin C deficiency is related to PD [27–29]. Vitamin C has been examined in several *in vitro* and *in vivo* models of PD and exhibited positive effects [30]. For instance, vitamin C was reported to have a neuroprotective effect against glutamate-mediated excitotoxicity in an *in vitro* model of human DA neurons [31]. In *Drosophila* model expressing human *α*-synuclein in the neurons, vitamin C treatment led to a delay in the loss of climbing ability [32].

Ubiquitin carboxyl-terminal hydrolase L1 (UCH-L1) is a member of deubiquitinating enzyme (DUB) family which plays an essential role in ubiquitin-proteasome system (UPS). Specially, UCH-L1 presents abundantly in brain and is comprised of 1–5% of total neuronal proteins [33]. The first evidence for the involvement of UCH-L1 in PD was the identification of a missense mutation in UCH-L1 (I93M) in a German family with PD [34]. Thereafter, several variants of UCH-L1 including E7A, R178Q, and A216D have been identified in patients with neurodegenerative disorders [35, 36]. In *Drosophila*, ubiquitin carboxyl-terminal hydrolase (dUCH) encoded by the *CG4265* gene is a homolog of human UCH-L1, sharing 43.7% identity [37]. Flies with specific knockdown of *dUCH* in DA neurons exhibited PD-like phenotypes including progressive decreases in not only locomotor ability, but also the number of DA neurons [37, 38]. Moreover, *dUCH* knockdown induced oxidative stress while curcumin, an antioxidant, decreased ROS levels and improved PD-like phenotypes induced by this knockdown [39]. The previous study also showed that vitamin C at the concentration of 0.5 mM reduced locomotive defects and the loss of DA neurons in larval stage caused by DA neuron-specific knockdown of *dUCH* [37].

In present study, we used the *Drosophila* model to examine the dose- and time-dependent effects of vitamin C treatment on PD-like phenotypes caused by knockdown of *dUCH*. We found that vitamin C had the potential to improve PD-like phenotypes which are characterized by locomotive defects and neurodegeneration in *dUCH* knockdown flies. However, vitamin C at high concentrations also exerted the negative effects on feeding behavior and locomotive ability. Noticeably, long-term treatment with vitamin C could protect against the degeneration of DA neuron, but led to a decrease in climbing ability of adult flies. Our results suggested that high dose of vitamin C and long-term treatment with this antioxidant might result in side effects on physiology.

## 2. Materials and Methods

### 2.1. Fly Stocks and Food Preparation

Fly stocks were reared on standard food containing 1% agar, 5% sucrose, 5% dry yeast, and 3% powdered milk at 25°C. The RNAi line carrying *UAS-dUCH.IR* (v26468, Vienna Drosophila Resource Center—VDRC) was used to knock down *dUCH*. The wild-type Canton-S (Bloomington Drosophila Stock Center—BDSC) was used to create control flies. The RNAi line was driven by *TH-GAL4* (*ple-GAL4*) (8848, BDSC) or *ELAV-GAL4* (8760, BDSC).

Vitamin C (L-Ascorbic acid, A0278, Sigma Aldrich, Singapore) was dissolved in distilled water and then added to standard food at final concentrations of 0.5 mM, 2.5 mM, and 5 mM. Vitamin C-containing food was kept in the dark to prevent oxidation during experimental procedures. Nutrient-restricted food contained low levels of nutrients which were reduced by 50% compared with those of standard food.

### 2.2. Feeding Assay

Feeding assay was performed as described previously [39] with some modifications. Food intake was measured indirectly by quantifying the amount of a dye larvae consumed. Coomassie Brilliant Blue G-250 dye (808274, MP Biochemicals, USA) was added to fly food at a concentration of 2%. Early third instar larvae were collected using a 20% sucrose solution and washed with PBS. These larvae were then transferred into food containing Coomassie dye and vitamin C at different concentrations and allowed to eat for 30 minutes. The larvae were washed with 70% ethanol and homogenized in 70% ethanol. The homogenates were then centrifuged at 9279×*g* for 10 minutes. The quantities of Coomassie in the supernatants were measured at OD595. The data were analyzed with a one-way ANOVA and graphed using GraphPad Prism 6.01.

### 2.3. Body Weight and Size Measurement

Male larvae in the late third instar stage were collected randomly, washed with PBS to remove food traces, and transferred to tubes with a density of eight larvae per tube. We weighed each tube containing larvae with an analytical balance. These larvae were then fixed with 4% paraformaldehyde overnight. The larvae were captured by a digital camera (Olympus E-M10) and two-dimensional larval size was measured using ImageJ (NIH, USA). The data of larval body weight and size were analyzed with a one-way ANOVA and graphed using GraphPad Prism 6.01.

### 2.4. Crawling Assay

The crawling assay was performed as described previously [37]. Male larvae in the third instar stage were collected randomly after being fed vitamin C and washed with PBS to discard food traces. After that, larvae were transferred to 2% agar plates with a density of 3-4 larvae per plate. The movement of larvae was recorded by a digital camera for 60 seconds. The recorded videos were then converted into AVI files using a MOV to AVI converter (Pazera Jacek, Poland) and analyzed by ImageJ (NIH, USA) with the wrMTrck plugin (developed by Dr. Jesper Søndergaard Pedersen). The data of crawling speed were analyzed with a one-way ANOVA and graphed using GraphPad Prism 6.01.

### 2.5. Climbing Assay

The climbing assay was performed as described previously [40]. Forty newly eclosed adult male flies were collected and transferred to cylindrical tubes with a height of 15 cm and a diameter of 2 cm. The tubes were tapped five times to collect the flies in the bottom, and the movements of flies were recorded for duration of 1 minute. These procedures were repeated five times and recorded by a digital camera. In all climbing experiments, the height to which each fly climbed after 5 seconds was scored as follows: 0 (less than 2 cm), 1 (between 2 and 4 cm), 2 (between 4 and 6 cm), 3 (between 6 and 8 cm), 4 (between 8 and 10 cm), and 5 (more than 10 cm). The climbing assay was performed until all flies lost their locomotor abilities. The climbing index was collected from a repeated measurement having average climbing score roughly equivalent to the mean of data from five repeated measurements. The data of climbing index was analyzed with a two-way ANOVA and graphed using GraphPad Prism 6.01.

### 2.6. Quantification of Dopaminergic Neurons by Immunostaining

Dopaminergic neurons were detected by staining with antityrosine hydroxylase (anti-TH), an enzyme catalyzing the conversion of tyrosine to L-DOPA [41]. Brains from third instar larvae or adult flies were dissected in PBS and fixed with 4% paraformaldehyde for 20 minutes. After being washed by 0.3% PBS-T (PBS containing 0.3% Triton X-100), samples were blocked by blocking solution (PBS containing 0.15% Triton X-100 and 10% normal goat serum) at 25°C for 30 minutes. Tissues were then incubated with a diluted rabbit anti-TH (1 : 250, Millipore) in blocking solution at 4°C for 36 hours. After being washed, brains were incubated with a secondary antibody conjugated with Alexa 488 (1:500, Invitrogen) at 25°C for 2 hours. Tissues were then washed and mounted in Vectashield Mounting Medium (Vector Laboratories, Japan). Samples were observed using ECLIPSE NI–U (Nikon). The numbers of dopaminergic neurons were counted by Cell Counter plugin in ImageJ 1.49° and analyzed with a one-way ANOVA using GraphPad Prism 6.01.

## 3. Results

### 3.1. Food Intake Ability of Larvae Was Affected by Vitamin C Treatment in a Dosage-Dependent Manner

**Figure 1:**
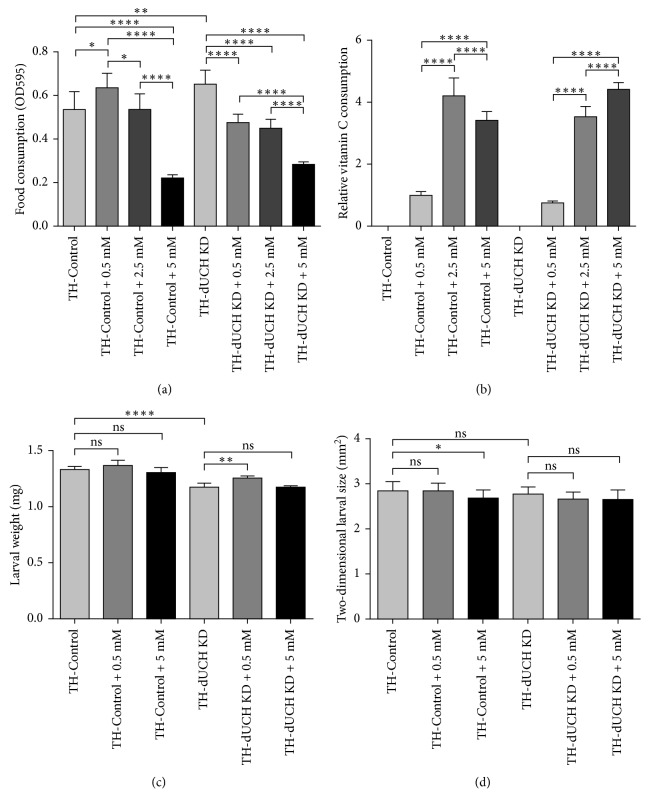
Vitamin C treatment affected the food intake ability of larvae in a dosage-dependent manner. (a) The levels of food intake were impacted by vitamin C treatment. There were changes in food intake ability when control and *dUCH* knockdown larvae were treated with different concentrations of vitamin C (one-way ANOVA with Tukey's test, ^*∗*^ *p* < 0.05, ^*∗∗*^ *p* < 0.01, and ^*∗∗∗∗*^ *p* < 0.0001, population size *N*=10 and biological replication *n*=7, error bars represent the standard deviation of data). (b) The relative levels of vitamin C intake. The differences in relative levels of vitamin C intake were statistically significant (one-way ANOVA with Tukey's test, ^*∗∗∗∗*^ *p* < 0.0001, population size *N*=10 and biological replication *n*=7, error bars represent the standard deviation of data). (c) The effects of vitamin C treatment on larval weight. The body weights of control and *dUCH* knockdown larvae were not reduced by vitamin C treatment (one-way ANOVA with Dunnett's test, ^*∗∗*^ *p* < 0.01 and ^*∗∗∗∗*^ *p* < 0.0001, population size *N*=8 and biological replication *n*=5, error bars represent the standard deviation of data). (d) The effects of vitamin C treatment on larval size. The body size of *dUCH* knockdown larvae were not reduced by vitamin C treatment (one-way ANOVA with Dunnett's test, ^*∗*^ *p* < 0.05, *n*=15, error bars represent the standard deviation of data). TH-Control (+; +; *TH-GAL4*/+) and TH-dUCH KD (+; +; *TH-GAL4*/*UAS-dUCH.IR*).

Vitamin C can change the taste of food which may, in turn, affect the amount of consumed food and fly health. Therefore, we measured the food intake ability of control (+; +; *TH-GAL4*/+) and *dUCH* knockdown larvae (+; +; *TH-GAL4*/*UAS-dUCH.IR*) when they were treated with food containing vitamin C by performing feeding assay on larvae. Vitamin C was added to fly food at concentrations of 0.5 mM, 2.5 mM, and 5 mM. In the control larvae, there was an increase (19%) in the amount of consumed food when they treated with a low concentration of vitamin C (0.5 mM) compared with untreated larvae. However, when the vitamin C concentration increased to 2.5 mM, the food intake ability of control larvae decreased compared to that of larvae treated with 0.5 mM, and it was equivalent to that of untreated larvae. Notably, at the higher concentration of vitamin C (5 mM), larval eating ability of the control decreased by 60% compared to that of untreated control larvae (Figure 1(a)). Based on the amount of vitamin C-containing food consumed by larvae, we estimated the relative levels of vitamin C intake. The data showed that control larvae consumed more levels of vitamin C (4.2-fold and 3.4-fold) at concentration of 2.5 mM and 5 mM, respectively, compared with 0.5 mM (Figure 1(b)). *dUCH* knockdown larvae were likely more sensitive to vitamin C than control larvae. When treated with vitamin C, their food intake ability considerably declined. The amount of food consumed by knockdown larvae decreased by 27% at 0.5 mM and 57% at 5 mM compared with that of untreated knockdown larvae (Figure 1(a)). The relative levels of vitamin C consumed by knockdown larvae were 4.7-fold and 5.9-fold higher at the concentration of 2.5 mM and 5 mM, respectively, compared with 0.5 mM (Figure 1(b)). The results indicated that Vitamin C treatment affected the food intake ability of larvae in a dosage dependent manner.

We found that feeding behavior of larvae was affected by vitamin C treatment. The decreases in the amount of food consumed by larvae in the early third instar stage might lead to nutritional deficiencies and reductions in larval weight and size. Therefore, we then evaluated the body weight and size of control and *dUCH* knockdown larvae when they were fed food containing vitamin C. The result showed that there were not significant differences in the weights of vitamin C-treated and untreated control larvae (Figure 1(c)). The size of control larvae reduced slightly at the concentration of 5 mM (Figure 1(d)). In *dUCH* knockdown larvae, vitamin C treatment at 0.5 mM caused a slight increase in larval weight compared with that of untreated knockdown larvae. At the higher concentration of vitamin C (5 mM), no significant difference was observed in the weights of treated and untreated knockdown larvae (Figure 1(c)). In addition, the sizes of knockdown larvae did not change significantly when they were treated with vitamin C (Figure 1(d)). The data indicated that the body weight and size of *dUCH* knockdown larvae were not reduced by vitamin C treatment, suggesting that knockdown larvae might meet their nutritional requirements.

### 3.2. Dose-Dependent Effects of Vitamin C on Crawling Abilities of *dUCH* Knockdown Larvae

**Figure 2:**
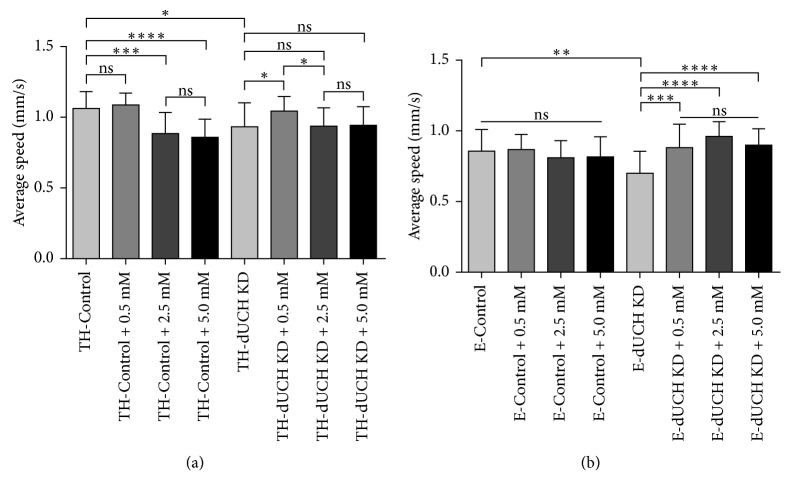
The influences of vitamin C treatment on larval crawling speed. (a) Average crawling speeds of larvae with specific knockdown of *dUCH* in dopaminergic neurons. Crawling ability of *dUCH* knockdown larvae was increased by treatment with low-dose of vitamin C (0.5 mM). However, there were negative effects on locomotive abilities when larvae treated with higher doses of vitamin C (one-way ANOVA with Kruskal–Wallis test and Dunn's test, ^*∗*^ *p* < 0.05, ^*∗∗∗*^ *p* < 0.001, and ^*∗∗∗∗*^ *p* < 0.0001, *n*=26, error bars indicate the standard deviation of data). (b) Average crawling speeds of larvae with specific knockdown of *dUCH* in pan-neurons. Vitamin C treatment also improved crawling ability of larvae with knockdown of *dUCH* in pan-neurons, while crawling speeds of control larvae were not affected by this treatment (one-way ANOVA with Kruskal–Wallis test and Dunn's test, ^*∗∗*^ *p* < 0.01, ^*∗∗∗*^ *p* < 0.001, and ^*∗∗∗∗*^ *p* < 0.0001, *n*=35, error bars indicate the standard deviation of data). TH-Control (+; +; *TH-GAL4*/+), TH-dUCH KD (+; +; *TH-GAL4*/*UAS-dUCH.IR*), E-Control (+; +; *ELAV-GAL4*/+), and E-dUCH KD (+; +; *ELAV-GAL4*/*UAS-dUCH.IR*).

*Drosophila* model with specific knockdown *dUCH* in DA neurons exhibited locomotive defects and vitamin C at 0.5 mM improved impaired crawling induced by this knockdown [37]. Therefore, we then examined the effects of vitamin C at different concentrations on crawling ability of *dUCH* knockdown flies. We found that knockdown of *dUCH* in DA neurons caused a decrease (0.13 mm/s) in crawling speed, which is consistent with previous finding [37]. Vitamin C at 0.5 mM did not affect the crawling ability of control larvae; however, crawling speeds of these larvae decreased when they were treated with higher concentrations of vitamin C (2.5 mM and 5 mM). There were decreased by 0.18 mm/s and 0.20 mm/s in crawling speeds of control larvae treated with 2.5 mM and 5 mM vitamin C, respectively, compared with untreated control larvae. In *dUCH* knockdown larvae, 0.5 mM vitamin C improved the locomotive defects caused by DA neuron-specific knockdown of *dUCH*. Nevertheless, there were not significant differences in crawling speeds between untreated and vitamin C-treated knockdown larvae at the concentrations of 2.5 mM and 5 mM (Figure 2(a)). These results suggested that the effects of vitamin C were dependent on dosage. Vitamin C at low concentration could increase the crawling abilities. By contrast, vitamin C exhibited side effects when larvae were treated with higher concentrations.

To further confirm the effects of vitamin C, we also performed crawling assay on larvae with knockdown of *dUCH* in pan-neurons using *ELAV-GAL4* driver. In this case, more neurons were affected by knockdown of *dUCH*. Similar to specific knockdown of *dUCH* in DA neurons, this knockdown in pan-neuron also resulted in a decrease (0.15 mm/s) in the crawling ability of *dUCH* knockdown larvae (+; +; *ELAV-GAL4*/*UAS-dUCH.IR*) compared with that of control larvae (+; +; *ELAV-GAL4*/+). No significant differences were observed in the crawling speeds of control larvae treated with different concentrations of vitamin C. In knockdown larvae, vitamin C treatment improved locomotive defects induced by pan-neuron-specific knockdown of *dUCH*. However, the crawling speeds of knockdown larvae were not significantly different among the concentrations of 0.5 mM, 2.5 mM, and 5 mM (Figure 2(b)). These results provided more evidence to demonstrate that vitamin C treatment could rescue locomotive impairments caused by *dUCH* knockdown.

### 3.3. Vitamin C Decreased the Loss of Dopaminergic Neurons in *dUCH* Knockdown Larvae

**Figure 3:**
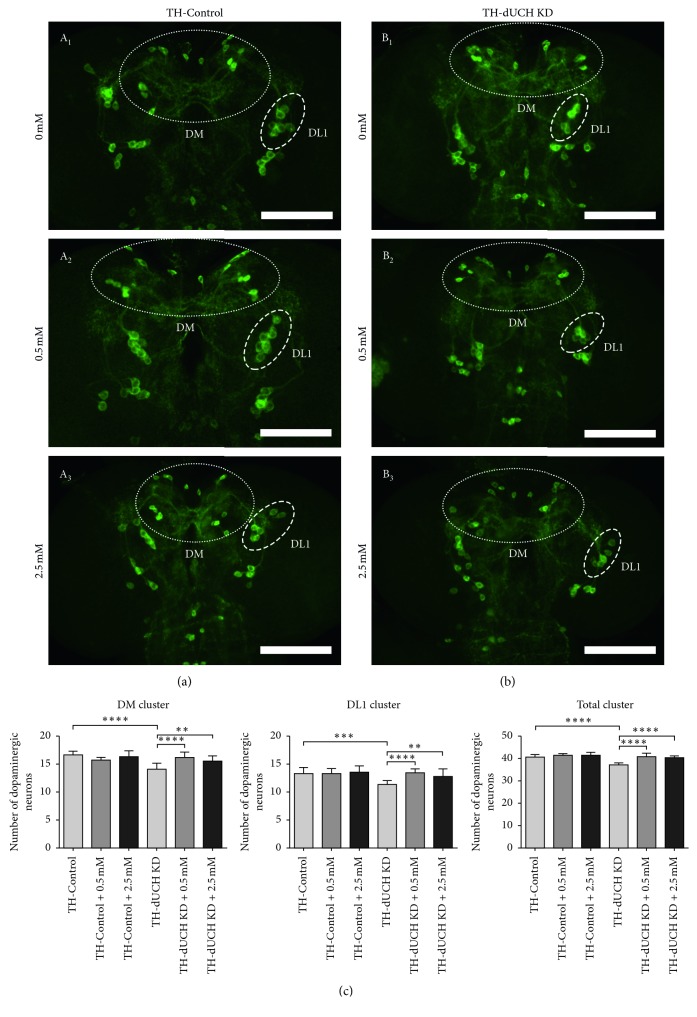
Vitamin C decreased the loss of dopaminergic neurons in *dUCH* knockdown larvae. Immunostaining images of brain lobes from control (TH-Control) and *dUCH* knockdown larvae (TH-dUCH KD) using anti-TH antibody. The larvae were treated with different concentrations of vitamin C including 0 mM (A_1_−B_1_), 0.5 mM (A_2_−B_2_), and 2.5 mM (A_3_−B_3_). DM and DL1 clusters are enclosed with white dashed ellipses and scale bars indicate 100 *µ*m. (C) Quantification of dopaminergic neurons in control and *dUCH* knockdown larvae treated with vitamin C Knockdown of *dUCH* caused decreases in the numbers of DA neurons in the DM, DL1 clusters, and all of clusters, while the loss of DA neurons in *dUCH* knockdown larvae was reduced by vitamin C treatment with 0.5 mM and 2.5 mM (one-way ANOVA with Sidak's test, ^*∗∗*^ *p* < 0.01, ^*∗∗∗*^ *p* < 0.001, and ^*∗∗∗∗*^ *p* < 0.0001, *n*=11, error bars indicate the standard deviation of data). TH-Control (+; +; *TH-GAL4*/+) and TH-dUCH KD (+; +; *TH-GAL4*/*UAS-dUCH.IR*).

The loss of DA neurons in the substantia nigra is a hallmark of PD, and *dUCH* knockdown also led to DA neuron degeneration in the *Drosophila* model [37, 42]. Therefore, we then evaluated the effects of vitamin C on the number of DA neurons at the larval stage. Based on the results of the crawling assay, the concentrations of 0.5 mM and 2.5 mM were chosen for performing the experiment to quantify DA neurons. Anti-TH (tyrosine hydroxylase) antibody was used to mark and visualize DA neurons. We also found that DA neuron-specific knockdown of *dUCH* led to decreases in the numbers of DA neurons. *dUCH* knockdown larvae had fewer DA neurons in the DM, DL1, and all of clusters (14, 11, and 37 neurons, respectively) than control larvae (17, 13, 41 neurons, respectively) (A_1_,B_1_, and C in Figure 3). The number of DA neurons in control larvae was not affected by vitamin C treatment. In *dUCH* knockdown larvae, the loss of DA neurons was decreased by vitamin C treatment with 0.5 mM and 2.5 mM. The numbers of DA neurons in DM, DL1, and all of clusters were higher in knockdown larvae treated with 0.5 mM vitamin C (16, 13, and 41 neurons, respectively) (B_2_ and C in Figure 3) than in vitamin C-untreated knockdown flies (B_1_ and C in Figure 3) and were similar to those in control larvae (A_1_ and C in Figure 3). Similarly, vitamin C at 2.5 mM also prevented against the loss of DA neurons induced by knockdown of *dUCH* (A, B_1_, B_3_, and C in Figure 3). The results suggested that vitamin C treatment could decrease the loss of DA neurons in the larval stage. This might lead to the improvement in crawling ability of *dUCH* knockdown larvae at low concentration of vitamin C (0.5 mM). At the concentration of 2.5 mM, although vitamin C reduced DA neuron loss, the crawling ability of knockdown larvae did not improve, suggesting that there might be other adverse effects of vitamin C at higher concentrations. Interestingly, we found that larvae fed nutrient-restricted food also exhibited a decrease in DA neuron degeneration (Figure S1). However, we demonstrated that vitamin C treatment with 0.5 mM and 5 mM did not cause nutrient deficiencies in *dUCH* knockdown larvae. These data proposed that the effects of vitamin C on PD-like phenotypes were not mediated through starving effects.

### 3.4. Vitamin C at 0.5 mM Decreased the Degeneration of Dopaminergic Neurons in *dUCH* Knockdown Flies over the Course of Aging

**Figure 4:**
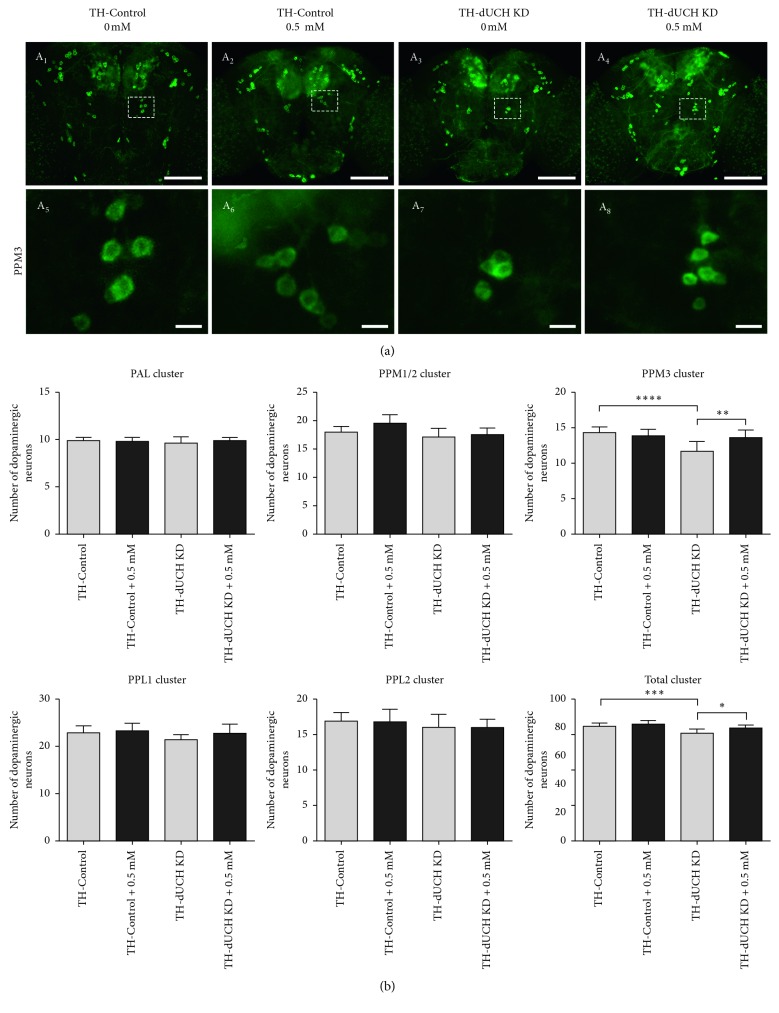
The effects of vitamin C at 0.5 mM on the numbers of DA neurons in 1-day-old flies. (a) Immunostaining images of brains from control (TH-Control) and *dUCH* knockdown flies (TH-dUCH KD) using anti-TH antibody. PPM3 clusters are enclosed by white dashed boxes and shown with a higher magnification in lower panel (A_5_–A_8_). Scale bars indicate 100 *µ*m (A_1_–A_4_) and 10 *µ*m (A_5_–A_8_). (b) Quantification of dopaminergic neurons in control and *dUCH* knockdown flies at 1 day. One-way ANOVA with Tukey's test, ^*∗*^ *p* < 0.05, ^*∗∗*^ *p* < 0.01, ^*∗∗∗*^ *p* < 0.001, and ^*∗∗∗∗*^ *p* < 0.0001, *n*=10, and error bars indicate the standard deviation of data. TH-Control (+; +; *TH-GAL4*/+) and TH-dUCH KD (+; +; *TH-GAL4*/*UAS-dUCH.IR*).

**Figure 5 fig5:**
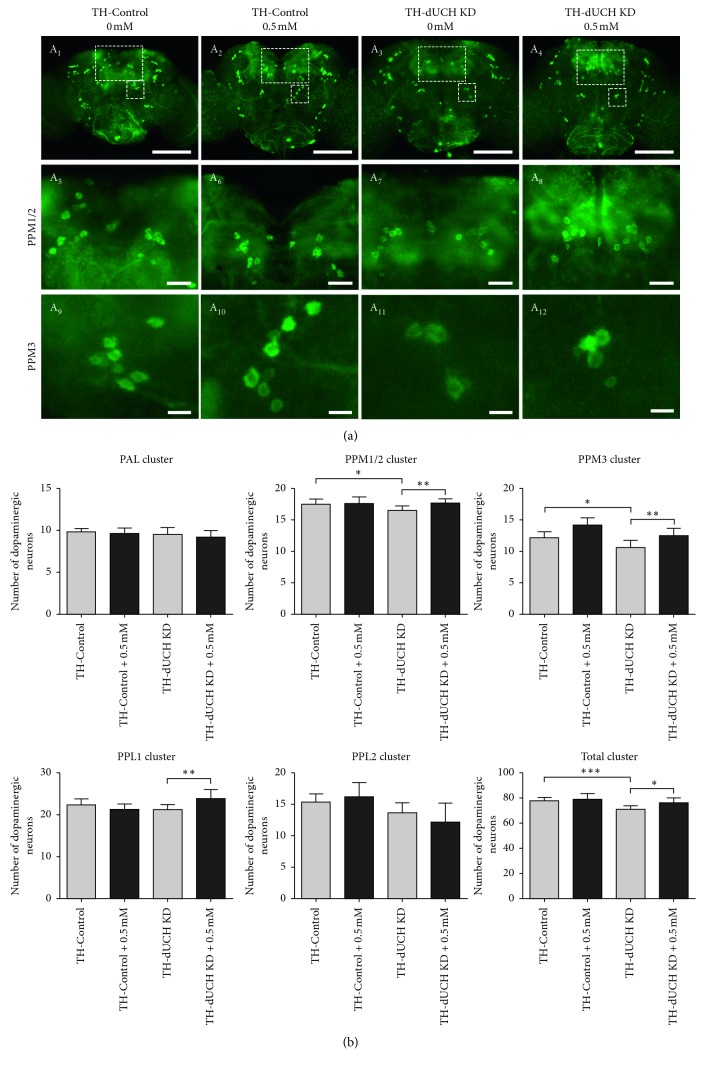
The effects of vitamin C at 0.5 mM on the number of DA neurons in 15-day-old flies. (a) Immunostaining images of brains from control (TH-Control) and *dUCH* knockdown flies (TH-dUCH KD) using anti-TH antibody. PPM1/2 and PPM3 clusters are enclosed by white dashed boxes and shown with higher magnifications in lower panels (A_5_–A_8_ and A_9_–A_12_, respectively). Scale bars indicate 100 *µ*m (A_1_–A_4_), 20 *µ*m (A_5_–A_8_), and 10 *µ*m (A_9_–A_12_). (b) Quantification of dopaminergic neurons in control and *dUCH* knockdown flies at 15 days. One-way ANOVA with Tukey's test, ^*∗*^ *p* < 0.05 and ^*∗∗*^ *p* < 0.01 and ^*∗∗∗*^ *p* < 0.001, *n*=10, and error bars indicate the standard deviation of data. TH-Control (+; +; *TH-GAL4*/+) and TH-dUCH KD (+; +; *TH-GAL4*/*UAS-dUCH.IR*).

**Figure 6 fig6:**
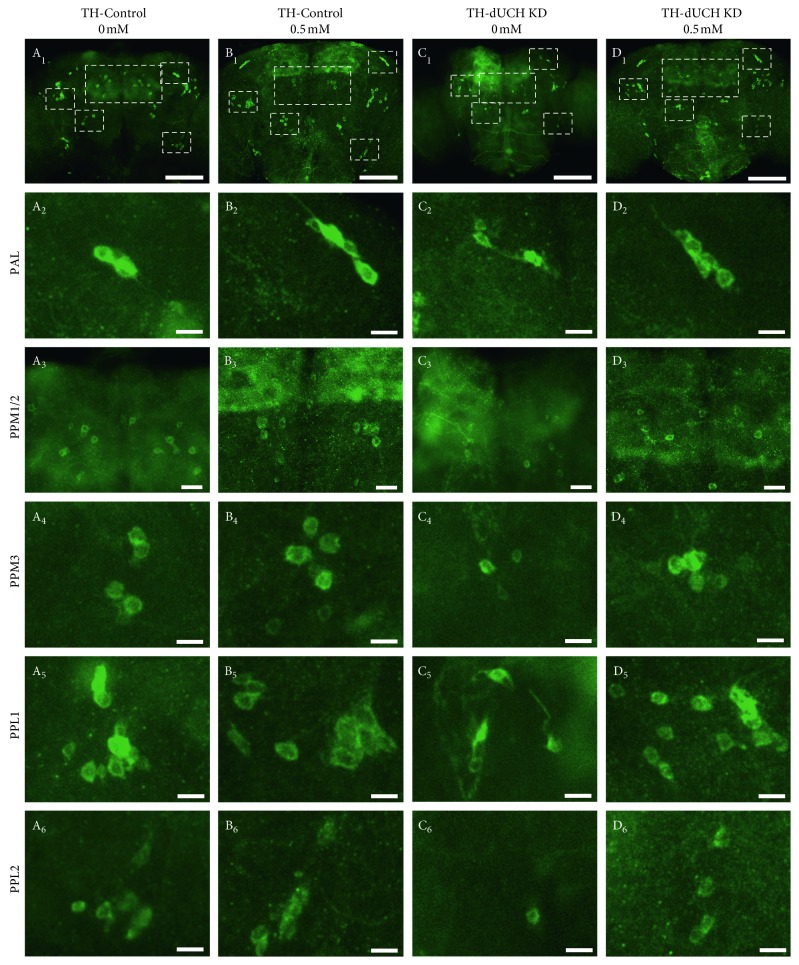
The effects of vitamin C at 0.5 mM on the number of DA neurons in 30-day-old flies. Immunostaining images of brains from control (TH-Control) and *dUCH* knockdown flies (TH-dUCH KD) using anti-TH antibody. The whole central brains were shown in A_1_–D_1_. PAL, PPM1/2, PPM3, PPL1, and PPL2 clusters are enclosed by white dashed boxes and shown with higher magnifications in lower panels (A_2_–D_2_, A_3_–D_3_, A_4_–D_4_, A_5_–D_5_, and A_6_–D_6_, respectively). Scale bars indicate 100 *µ*m (A_1_–D_1_), 20 *µ*m (A_3_–D_3_), and 10 *µ*m (A_2_–D_2_, A_4_–D_4_, A_5_–D_5_, and A_6_–D_6_). TH-Control (+; +; *TH-GAL4*/+) and TH-dUCH KD (+; +; *TH-GAL4*/*UAS-dUCH.IR*).

**Figure 7:**
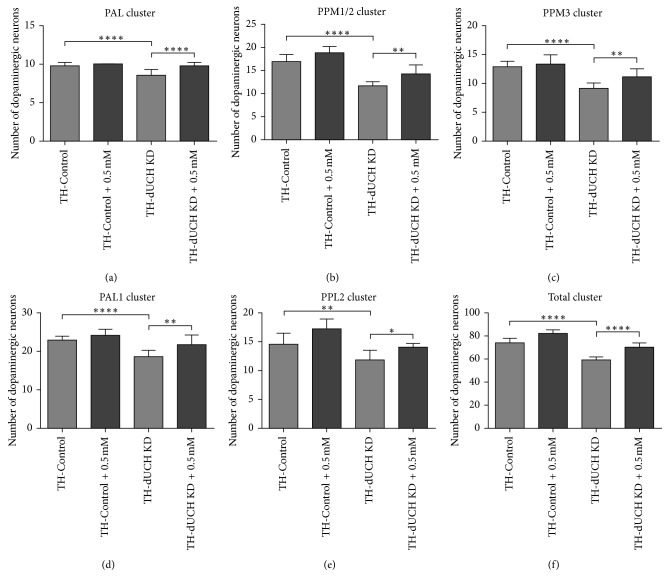
Vitamin C at 0.5 mM decreased the degeneration of dopaminergic neurons in *dUCH* knockdown flies at 30 days old. The numbers of DA neurons in 30-day-old brains shown in Figure 6 were quantified and statistically analyzed (one-way ANOVA with Dunnett's test, ^*∗*^ *p* < 0.05, ^*∗∗*^ *p* < 0.01, and ^*∗∗∗∗*^ *p* < 0.0001, *n*=9, and error bars indicate the standard deviation of data). TH-Control (+; +; *TH-GAL4*/+) and TH-dUCH KD (+; +; *TH-GAL4*/*UAS-dUCH.IR*). (a) PAL cluster, (b) PPM1/2 cluster, (c) PPM3 cluster, (d) PPL1 cluster, (e) PPL2 cluster, and (f) Total cluster.

In PD patients, the degeneration of DA neuron is progressive over the course of aging [5]. Flies with DA neuron-specific knockdown of *dUCH* also exhibited a progressive loss of DA neurons [37]. Therefore, we then evaluated the effects of vitamin C on the number of DA neurons in adult flies at day 1, 15, and 30. The concentration of 0.5 mM vitamin C was chosen for this experiment based on the most effective improvements in PD-like phenotypes of *dUCH* knockdown larvae treated with this concentration. The results showed that there were decreases in the number of DA neurons and the level of neurodegeneration increased with age (Figures 4 [Fig fig5] [Fig fig6]–7). The number of clusters exhibiting the loss of DA neurons increased from day 1 to day 30. At day 1, the higher level of DA neuron degeneration in *dUCH* knockdown flies compared with control flies occurred in merely one cluster (PPM3) (Figure 4). At day 15, there were two clusters (PPM1/2 and PPM3) experiencing the higher levels of DA neuron loss in *dUCH* knockdown flies (Figure 5). In 30-day-old flies with knockdown of *dUCH*, the number of clusters exhibiting the higher level of DA neuron degeneration was increased to five (PAL, PPM1/2, PPM3, PPL1, PPL2, and PPL2) (Figures 6 and 7). However, vitamin C treatment decelerated the progression of neurodegeneration induced by *dUCH* knockdown in DA neuron. *dUCH* knockdown flies treated with vitamin C had higher numbers of DA neurons in PPM3 cluster at day 1, PPM1/2, PPM3, and PPL1 at day 15, and all five cluster at day 30 than untreated knockdown flies. Furthermore, there were no significant differences in the number of DA neurons between vitamin C-treated knockdown flies and untreated control flies at all examined times (Figures 4 [Fig fig5] [Fig fig6]–7). These results suggested that vitamin C could protect against the degeneration of DA neurons induced by *dUCH* knockdown over the course of aging.

### 3.5. Long-Term Consumption of Vitamin C Caused Decreases in Climbing Abilities of *dUCH* Knockdown Flies

**Figure 8:**
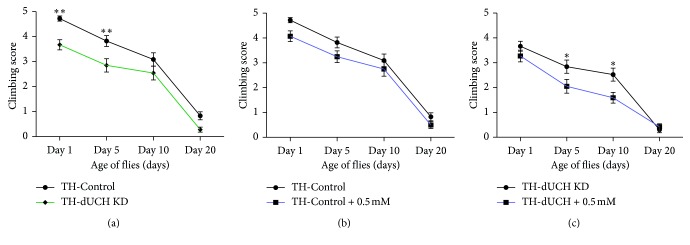
The influences of vitamin C treatment on adult climbing ability. (a) The knockdown of *dUCH* in dopaminergic neurons caused a decline in climbing ability. (b) Vitamin C did not affect climbing ability of control flies. (c) *dUCH* knockdown flies treated with vitamin C at concentration of 0.5 mM exhibited decreases in locomotive ability. Two-way ANOVA with Sidak's test, ^*∗*^ *p* < 0.05 and ^*∗∗*^ *p* < 0.01, *n*=40, error bars indicate the standard error of the mean. TH-Control (+; +; *TH-GAL4*/+) and TH-dUCH KD (+; +; *TH-GAL4*/*UAS-dUCH.IR*).

The above-mentioned data demonstrated that vitamin C at 0.5 mM could decrease the degeneration of DA neurons in adult flies, suggesting that locomotive ability of *dUCH* knockdown flies would improve when treated with 0.5 mM vitamin C. Therefore, we then performed climbing assay to investigate the effects of long-term treatment with 0.5 mM vitamin C on locomotive ability. The results showed there were decreases in climbing ability of *dUCH* knockdown flies compared with control flies (Figure 8(a)). Vitamin C treatment with concentration of 0.5 mM did not affect the mobility of control flies (Figure 8(b)). However, *dUCH* knockdown flies were more sensitive to vitamin C than control larvae and exhibited decreases in climbing ability when treated with vitamin C. At day 1 after eclosion, there was no significant difference in mobility between treated and untreated knockdown flies. At day 5 and day 10, vitamin C-treated knockdown flies exhibited less mobility than untreated knockdown flies (Figure 8(c)). The results suggested that in addition to neuroprotective effects, long-term treatment of vitamin C might also exert the side effects on fly physiology.

## 4. Discussion

Parkinson's disease is one of the most common neurodegenerative disorders worldwide [1]. However, current therapies merely support to control PD symptoms but not cure this disease [43, 44]. The increasing evidence for the involvement of oxidative stress in neurodegenerative diseases including PD has been reported [45–47]. Therefore, antioxidants are considered as a promising therapy [48].

Previous studies reported that *Drosophila* model with DA neuron-specific knockdown of *dUCH*, a homolog of *UCH-L1*, exhibited PD-like phenotypes which are characterized by progressive locomotive impairments, DA neurodegeneration, and depletion of dopamine [37]. Furthermore, a recent study provided the evidence that knockdown of *dUCH* induced intracellular ROS levels in larval eye imaginal discs and adult brains [39]. Antioxidants such as curcumin (1 mM) and vitamin C (0.5 mM) could protect against the locomotive defects and neurodegeneration induced by this knockdown [37, 39]. These suggested *dUCH* knockdown fly is a potential model for screening antioxidants to treat PD. Therefore, in this study, we used flies model with knockdown of *dUCH* to evaluate the dose- and time-dependent effects of vitamin C treatment on PD-like phenotypes.

Vitamin C is one of essential nutritional antioxidants and plays many important functions in human body, especially in brain. The biological functions of vitamin C include antioxidant, cofactor of enzyme, and neuromodulator [25, 49]. Humans have to obtain vitamin C through food and supplements due to inability to synthesize vitamin C [24]. Several studies have reported that vitamin C deficiency is related to PD, and dietary vitamin C intake reduced the risk of PD [27–29,50]. Furthermore, L-Dopa absorption was improved in elderly PD patients when they received cotreatment of vitamin C [51]. Besides that, vitamin C had been investigated in several PD models and exhibited positive effects. In *in vitro* model of human DA neurons, vitamin C could prevent against cell death induced by glutamate [31]. Vitamin C treatment could also delay the loss of climbing ability in human *α*-synuclein-expressing fly model [32]. However, only a few studies did not provide sufficient evidence of the potential of vitamin C for PD treatment.

Our results showed that vitamin C improved crawling ability and the loss of DA neurons in *dUCH* knockdown larvae. Vitamin C at 0.5 mM could improve crawling ability of knockdown larvae; however, there were not improvements in locomotive ability at higher concentrations (2.5 mM and 5 mM). Moreover, although long-term treatment with vitamin C at 0.5 mM could improve the progressive degeneration of DA neurons, this treatment led to decrease in climbing ability in the adult stage. The result suggested that in addition to the neuroprotective effects, vitamin C residues might impact fly physiology. This hypothesis was confirmed by the results from crawling assay on larvae with specific knockdown of *dUCH* in pan-neurons. In larvae with pan-neuron-specific knockdown of *dUCH*, crawling ability could be improved by vitamin C treatment and the degree of improvements was not different among all examined concentrations. This proposed that more neurons were influenced by knockdown of *dUCH*; therefore, more amount of vitamin C might be required to decrease the effect of this knockdown, leading to decreases in level of vitamin C residues and side effects. In addition, vitamin C also affected the feeding behavior of larvae in the early third instar stage, leading to decreases in the food intake abilities of control (at 5 mM) and *dUCH* knockdown larvae (at 0.5 mM, 2.5 mM, and 5 mM).

The side effects of vitamin C have been also observed in humans. Normally, the dietary recommendations (RDA) of vitamin C are 90 mg/day and 75 mg/day for adult men and women, respectively (the U.S. Institute of Medicine, IOM) [52]. The consumption of high level of vitamin C has been reported to have adverse effects on physiology [53]. For example, administration of massive doses of vitamin C led to renal failure and oxalate nephropathy [54, 55]. Furthermore, vitamin C can also act as a pro-oxidant which induces oxidative stress in pathological conditions [56]. These suggested that vitamin C not only has neuroprotective impacts but also exerts adverse effects when consuming the overdose of this compound. Therefore, it is necessary to consider dose-dependent effects of vitamin C on treating PD.

## Data Availability

All data of this study are available from the corresponding author upon reasonable request.

## Supplementary Materials

Supplementary Materials Figure S1: the effects of food restrictions on larval body weight and size and the number of DA neurons. *dUCH* knockdown larvae (TH-dUCH KD) were fed standard food (100% nutrients), standard food containing 0.5 mM vitamin C (100% nutrients + 0.5 mM vitC), or nutrient-restricted food (50% nutrients). (A) The body weight of *dUCH* knockdown larvae was reduced by 18% when they were fed nutrient-restricted food (one-way ANOVA with Dunnett's test, ^*∗*^ *p* < 0.05 and ^*∗∗∗∗*^ *p* < 0.0001, population size *N*=8 and biological replication *n*=5, error bars represent the standard deviation of data). (B) The body size of *dUCH* knockdown larvae was reduced 16% when they were fed nutrient-restricted food (one-way ANOVA with Kruskal–Wallis test and Dunn's test, ^*∗∗∗∗*^ *p* < 0.0001, *n*=15, error bars represent the standard deviation of data). (C) Immunostaining images of brain lobes from *dUCH* knockdown larvae using anti-TH antibody. DL1 clusters are enclosed with white dashed ellipses, and scale bars indicate 100 *µ*m. TH-dUCH KD (+; +; *TH-GAL4*/*UAS-dUCH.IR*).
