# Supplementary material for: Evaluating Dose- and Time-Dependent Effects of Vitamin C Treatment on a Parkinson's Disease Fly Model
Source: Parkinsons Dis. 2019 Jan 2;2019:9720546. doi: 10.1155/2019/9720546 (PMC6334328; doi:10.1155/2019/9720546)
Supplement: Supplementary Materials — Figure S1: the effects of food restrictions on larval body weight and size and the number of DA neurons. dUCH knockdown larvae (TH-dUCH KD) were fed standard food (100% nutrients), standard food containing 0.5 mM vitamin C (100% nutrients + 0.5 mM vitC), or nutrient-restricted food (50% nutrients). (A) The body weight of dUCH knockdown larvae was reduced by 18% when they were fed nutrient-restricted food (one-way ANOVA with Dunnett's test, ∗ p < 0.05 and ∗∗∗∗ p < 0.0001, population size N=8 and biological replication n=5, error bars represent the standard deviation of data). (B) The body size of dUCH knockdown larvae was reduced 16% when they were fed nutrient-restricted food (one-way ANOVA with Kruskal–Wallis test and Dunn's test, ∗∗∗∗ p < 0.0001, n=15, error bars represent the standard deviation of data). (C) Immunostaining images of brain lobes from dUCH knockdown larvae using anti-TH antibody. DL1 clusters are enclosed with white dashed ellipses, and scale bars indicate 100 µm. TH-dUCH KD (+; +; TH-GAL4/UAS-dUCH.IR). [file 9720546.f1.docx]

**Supplementary Materials**

**
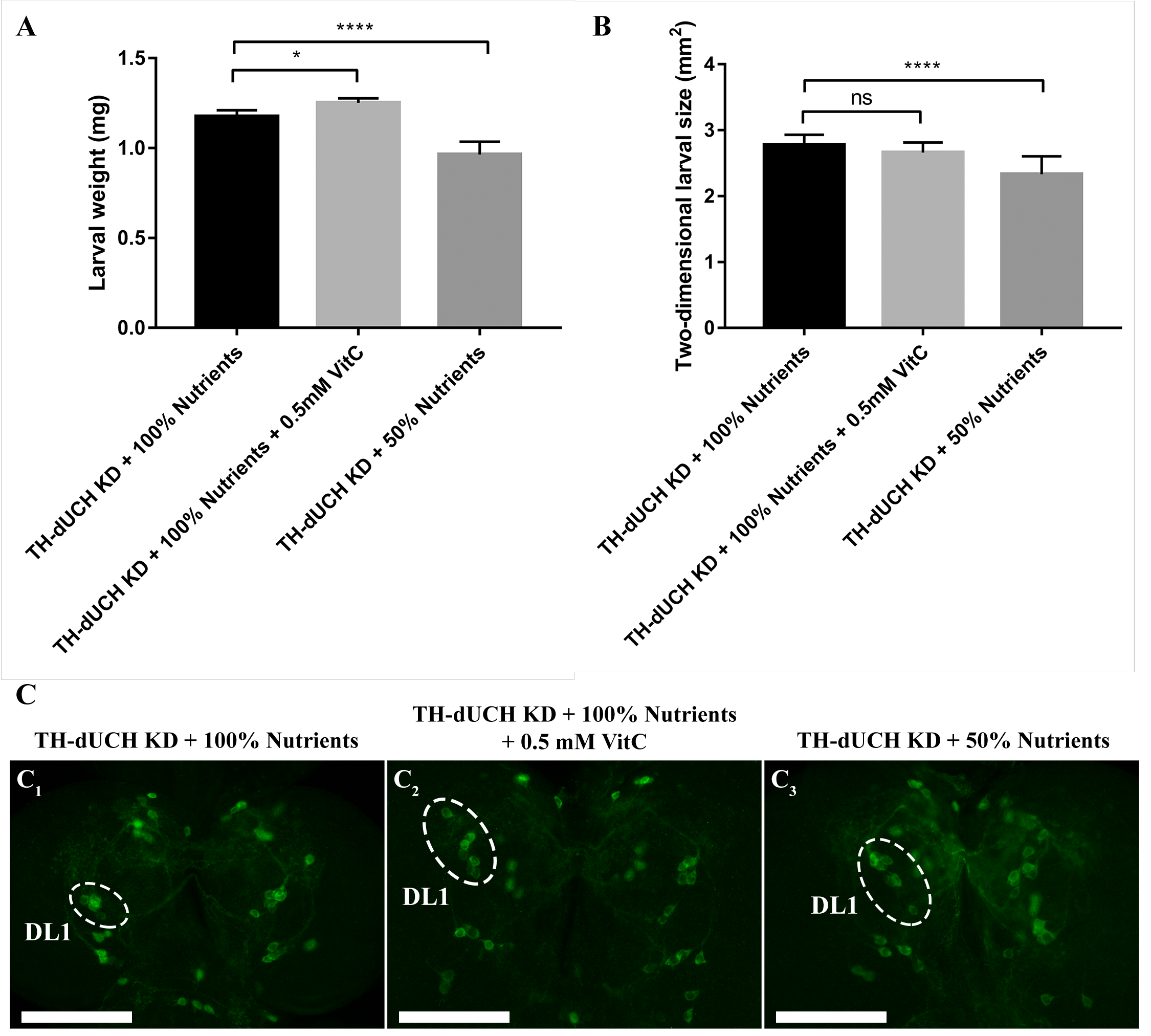
**

**Figure S1. The effects of food restrictions on larval body weight and size and the number of DA neurons.** *dUCH* knockdown larvae (TH-dUCH KD) were fed standard food (100% nutrients), standard food containing 0.5 mM vitamin C (100% nutrients + 0.5 mM vitC), or nutrient-restricted food (50% nutrients). (A) The body weight of *dUCH* knockdown larvae was reduced by 18% when they were fed nutrient-restricted food (One-way ANOVA with Dunnett's test, *p<0.05 and ****p<0.0001, population size N=8 and biological replication n=5, error bars represent the standard deviation of data). (B) The body size of *dUCH* knockdown larvae was reduced 16% when they were fed nutrient-restricted food (One-way ANOVA with Kruskal-Wallis test and Dunn's test, ****p<0.0001, n=15, error bars represent the standard deviation of data). (C) Immunostaining images of brain lobes from *dUCH* knockdown larvae using anti-TH antibody. DL1 clusters are enclosed with white dashed ellipses and scale bars indicate 100 µm. TH-dUCH KD (*+; +; TH-GAL4/UAS-dUCH.IR*).
